# Proposal of a framework for evaluating military surveillance systems for early detection of outbreaks on duty areas

**DOI:** 10.1186/1471-2458-8-146

**Published:** 2008-04-30

**Authors:** Jean-Baptiste Meynard, Herve Chaudet, Andrew D Green, Henry L Jefferson, Gaetan Texier, Daniel Webber, Bruce Dupuy, Jean-Paul Boutin

**Affiliations:** 1 Institut Pasteur de la Guyane, Avenue Pasteur, Cayenne, French Guiana; 2 Université de la Méditerranée, Boulevard Jean Moulin, Marseilles, France; 3 Defence Medical Services Department, London, UK; 4 Liverpool School of Tropical Medicine, Liverpool, UK; 5 Institut de Médecine Tropicale du service de santé des armées, Parc du Pharo, Marseilles, France; 6 Defence Science and Technology Laboratory, Porton Down, UK

## Abstract

**Background:**

In recent years a wide variety of epidemiological surveillance systems have been developed to provide early identification of outbreaks of infectious disease. Each system has had its own strengths and weaknesses. In 2002 a Working Group of the Centers for Disease Control and Prevention (CDC) produced a framework for evaluation, which proved suitable for many public health surveillance systems. However this did not easily adapt to the military setting, where by necessity a variety of different parameters are assessed, different constraints placed on the systems, and different objectives required. This paper describes a proposed framework for evaluation of military syndromic surveillance systems designed to detect outbreaks of disease on operational deployments.

**Methods:**

The new framework described in this paper was developed from the cumulative experience of British and French military syndromic surveillance systems. The methods included a general assessment framework (CDC), followed by more specific methods of conducting evaluation. These included Knowledge/Attitude/Practice surveys (KAP surveys), technical audits, ergonomic studies, simulations and multi-national exercises. A variety of military constraints required integration into the evaluation. Examples of these include the variability of geographical conditions in the field, deployment to areas without prior knowledge of naturally-occurring disease patterns, the differences in field sanitation between locations and over the length of deployment, the mobility of military forces, turnover of personnel, continuity of surveillance across different locations, integration with surveillance systems from other nations working alongside each other, compatibility with non-medical information systems, and security.

**Results:**

A framework for evaluation has been developed that can be used for military surveillance systems in a staged manner consisting of initial, intermediate and final evaluations. For each stage of the process parameters for assessment have been defined and methods identified.

**Conclusion:**

The combined experiences of French and British syndromic surveillance systems developed for use in deployed military forces has allowed the development of a specific evaluation framework. The tool is suitable for use by all nations who wish to evaluate syndromic surveillance in their own military forces. It could also be useful for civilian mobile systems or for national security surveillance systems.

## Background

Evaluation is a major issue for Public Health. Evaluation involves fundamental judgements concerning a particular intervention, based on a system providing scientifically valid and socially legitimate information about the intervention itself or its components [1]. Evaluation is particularly important for the new generation of surveillance systems. Since the 2001 attack on the World Trade Center and other terrorist attacks, considerable efforts have been made to develop syndromic surveillance systems, particularly with the US, but also in Europe. In the face of the heightened risk of bioterrorism, the main objective of these systems is to provide early warning of potential outbreaks, enabling the authorities to react rapidly. The importance of evaluating such systems quickly became apparent [2]. Several evaluations of such systems have been performed. However, these evaluations have rarely used validated and standardised methodologies. A framework for the evaluation of civilian syndromic surveillance systems was created by the Centers for Disease Control and Prevention (CDC) of Atlanta in 2003 [3] and revised in 2004 [4]. This framework was developed to evaluate whether such systems attain their objectives, and to provide information for further development and improvement.

Since the meeting of the North Atlantic Treaty Organisation (NATO) in 2002 [5], armed forces have also begun to develop syndromic surveillance systems, with their own objectives and procedures, adapted to missions and engagement conditions during operations. The main objectives of these systems are the early detection of potential epidemics, evaluation of their potential impact on operational capacity and the provision of information to facilitate the medical response. Military syndromic surveillance systems must take into account medical, technological, human and organisational aspects potentially very different from those considered in civilian systems.

Since 2002, the French (FR) armed forces have been developing a syndromic surveillance system, the *système de surveillance spatiale des épidémies au sein des forces armées en Guyane *(2SE FAG). ThIS prototype of syndromic surveillance was developed at the *Institut de Médecine Tropicale du service de santé des armées *(IMTSSA) in Marseilles, in collaboration with the *Université de la Méditerranée *in Marseilles and the *Institut Pasteur de la Guyane *in Cayenne. This system has been in use among French armed forces in French Guiana since 2004 [6].

After the first Gulf war in 1991, the UK armed forces began developing the Prototype Remote Illness and Symptom Monitor (PRISM). This system was implemented during employment of the UK contingent in Iraq in 2003, but the experiment was deemed a failure and the system was taken out of operation in 2004. Evaluation of this system led to the creation of a new system, the Real-time Medical Surveillance (RMS) system, which is currently being developed further.

These systems have provided invaluable insight into the specific aspects and requirements of military syndromic surveillance systems and their evaluation. Building on this experience, we developed a new method for evaluating military syndromic surveillance systems, which is described here.

The objective of this study was to describe a new framework for evaluating military surveillance systems for the early detection of outbreaks on duty areas.

## Methods

A specific evaluation method adapted to military syndromic surveillance objectives and conditions of engagement in the field was developed. This method was used to evaluate the FR 2SE FAG and UK PRISM and RMS systems.

### French and British military syndromic surveillance systems

2SE FAG (table 1) was set up in French Guiana to complement the mandatory surveillance system, which was used as the reference during the final evaluation of the system [7]. This system is based on two independent networks working together: a recording network, situated in French Guiana, and an analysis network, situated in both French Guiana and mainland France. The recording network is based on the input of health-related information into the system by general practitioners, nurses and paramedics. Various electronic input methods (PC, PDA, GPS and satellite communication tools) may be used, depending on the situation. The data is then analysed by an analysis network, the *communauté de services pour la surveillance syndromique *(CS^3^). This results in the production of automated dashboards which display health information to be used directly by commanders, defining automatically 3 levels of situation: normal situation, pre-alarm situation and alarm situation. This system is currently designed for use in fever surveillance, in an intertropical area affected by many febrile tropical diseases, including dengue fever and malaria. Right from the initial development of this system, an evaluation strategy has been used to evaluate its efficacy and to improve its functioning before its widespread implementation throughout the French armed forces.

**Table 1:** Main characteristics of the French (2SE FAG) and British (PRISM and RMS) military syndromic surveillance systems

	**2SE FAG**	**PRISM**	**RMS**
**Nationality**	French	British	British
**Year begun**	2002	1991	2005
**Year ended**	-	2004	-
**Recorded data for patients**	Specific list of symptoms *	Specific list of symptoms *	Specific list of symptoms *
**Person responsible for data input**	Military general practitioner or nurse	Military personnel (not from health service)	Military personnel (not from health service)
**Support for input**	PC or PDA **	PDA	PDA
**Data transmission**	Telephone link Communication satellite	Communication satellite	Communication satellite
**Automatization of data analysis**	Automated	Not automated	Not automated
**Method of data analysis**	CPEG, EWMA **	-	-
**Synthesis for commanders**	Real-time automated indicators	No indicators	Non-automated indicators
**Feed-back for actors**	In real time	-	Weekly
**Evaluation programme**	Before, during and after deployment	-	Before, during and after deployment

2SE FAG: "Système de surveillance en temps réel au sein des forces armées en Guyane"

PRISM: Prototype Remote Illness and Symptom Monitor

RMS: Real-time Medical Surveillance

* The specific lists of symptoms are different for the French and British systems

** PC: Personal computer, PDA: Personal digital assistant

*** CPEG: Current/past experience graph, EWMA: Exponential weighted moving average

PRISM (table 1), together with the use of PDA and satellite communication systems, was designed to facilitate the surveillance of many different types of symptom among military personnel: fever, cardiac, neurological, respiratory, gastrological and dermatological symptoms. Data were sent from the theatre of action to the analysis centre within the Defence Science and Technology Laboratory (DSTL) at Porton Down. These data were then integrated into a Geographical Information System (GIS) and statistical analysis was carried out in DSTL, generating results in a form easily accessible to commanders. It was necessary to define what constituted an alarm for this system. Despite its overall failure, the evaluation of PRISM paved the way for the development of RMS, which has yet to be formally evaluated.

### Functioning of military syndromic surveillance and evaluation parameters

**Figure 1:**
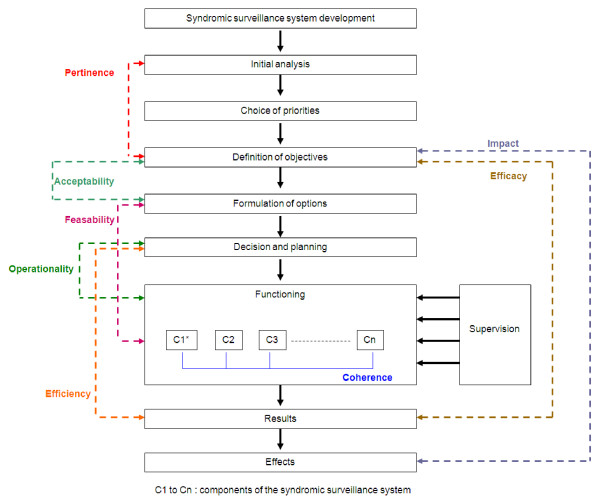
The development steps for a syndromic surveillance system and their evaluation parameters.

Several stages were necessary in the development and evaluation of military surveillance systems, as shown in figure 1. From the initial analysis to the results, several evaluation parameters in addition to those of the general CDC assessment framework were studied: 1) Pertinence – the link between objectives and the preliminary assessment; 2) Feasibility – the extent to which the available means meet needs; 3) Operationality – functioning conditions in the field; 4) Coherence – the link between the different components and the development stages; 5) Efficacy – the extent to which the initial objectives are achieved; 6) Efficiency – the link between the resources implemented and the results and 7) Impact – all effects other than the results.

**Figure 2:**
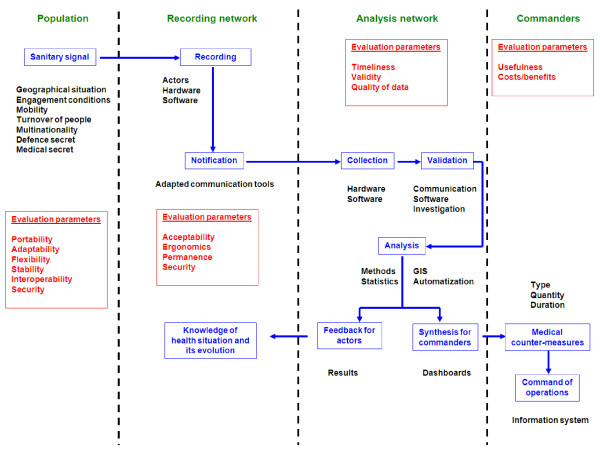
Epidemiological information circulation and use and the corresponding evaluation parameters during the functioning of a military syndromic surveillance system.

The functioning step was detailed, with careful study of the process of information circulation and use and of the corresponding evaluation parameters (figure 2). The following additional factors were also studied: 1) Timeliness, assessing the time taken to detect a potential epidemic and to provide commanders with the relevant information; 2) Validity, assessing the system's ability to detect real outbreaks; 3) Quality of data, providing information about the completeness of recorded data; 4) Usefulness, measuring the contribution of the system to the early detection of an epidemic and its ability to provide information to facilitate efficient intervention; 5) Flexibility, measuring the system's ability to adapt to a change in environmental conditions, such as the emergence of a disease causing an epidemic (emergent disease) or significant changes in the population; 6) Acceptability, assessing the willingness of users to be involved in the operation of the system; 7) Portability, evaluating the possible use of the system in other circumstances or at a different location; 8) Stability, reflecting the reaction of the system to a change in the variables recorded; 9) Financial assessment, dealing with installation and running costs and the calculation of cost-efficacy and cost-benefit ratios.

These evaluation parameters take into account the current constraints facing armed forces in conditions of engagement, and concern mostly variability in the geographical location of missions and the possibility of deployment in locations for which no sanitary data are available. The high mobility of forces within a theatre, the permanent turnover of soldiers, stress and difficult work conditions in the field, the multinational character of current missions, the necessity of the functioning system to be absolutely permanent and to continue to operate for long periods of time in many different areas, particularly at the start of deployment, were also important issues. Other issues included the diffuse geographical distribution of those involved in surveillance, compatibility between the surveillance system and the information systems used by commanders and the high level of security required for both military and medical data.

### Other military characteristics and core evaluation methods

**Figure 3:**
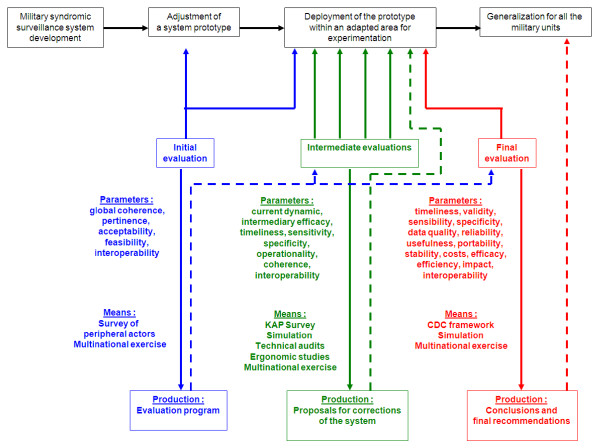
Schematic representation of the proposed framework for evaluating military surveillance systems for the early detection of outbreaks on tours of duty.

The evaluation method for military systems was developed around the three steps classically used in evaluation (figure 3) [8]. The initial evaluation ensures that the system has a solid basis and is entirely coherent. It leads to the creation of an evaluation program, for which it conditions the modes of decision-making, follow-up and overall activities. Intermediate evaluations are required to monitor and correct actions. These evaluations check the dynamics of actions, reorienting them towards their initial objectives. Final evaluation assesses the system and its effects, making it possible to analyse, interpret and use the results and providing an appraisal of the extent to which the system was successful and data for current and future enhancement of the system.

We focused on technical aspects. The development of a secure architecture for the recording, notification and analysis of information is one of the first problems that must be tackled for any new system. This architecture encompasses the entire surveillance process, from data acquisition to the issuing of an alarm. For military systems, maximum flexibility must be combined with the highest level of security. It must also be possible to adapt the capacity of the system to demand, maintaining some redundancy so that the system can be modified whilst still operational. Technical audits, based on quality assurance methods[9], were used to study functioning and security. The permanence of the system is a key requirement and evaluation. Regular intrusion tests were performed and we also tried to block the system by supplying it with excessive amounts of data.

We also assessed the ergonomics of the system, taking into account all modes of intervention, with adaptation to different types of armed forces and deployment. Several types of study were used to study ergonomics, and these studies concerned both hardware and software. The focus group method was used for the analysis of tasks and activities [10]. The cognitive walkthrough method [11] was used for precise analysis of the actions taken. In this analysis, the reactions of new users of the system were analysed, together with their opinions. Software interfaces were analysed (size, colour and type of character, drop-down menus, etc.) with the Bastien, Scapin and Nogier criteria [12]. The UML method [13] was also used to formalize and to describe each task and the overall activity of the system precisely.

We also carried out a specific evaluation of the users of the recording network. Even if the architecture of the system is technologically successful, a surveillance system works only if the users of the system have understood the issues involved and the objectives of the system, know how to use the tools to carry out their tasks and periodically receive significant feedback. Knowledge, attitudes and practice surveys (KAP) were used for this evaluation. This type of survey can be used to evaluate factors following a specific questionnaire and face-to-face interviews with all or a sample of stakeholders. The questionnaire was tested in a pilot study, on a small sample of the population before the study began.

The actors of the evaluation were some actors of the system, university or institutional partners or came from external organisms. They were military and civilian. They were specialists in epidemiological surveillance or its evaluation.

The CDC reference framework [4] was used for the evaluation of 2SE FAG. This framework explores a number of different areas and performances in different functions. The first part of the survey involves a description of the system, its objectives and operational aspects and a detailed presentation of the stakeholders. The second part evaluates the potential of the system to detect an outbreak. The third part assesses the experiences of the users of the system. This approach required the creation of a database and of a specific questionnaire for 2SE FAG stakeholders.

The timeliness of the systems was assessed by simulating an outbreak of aggressive or natural biological agents. These techniques were applied to the French and British systems separately, but were also applied to these two systems used together during a NATO exercise, with a simulation of dysentery and anthrax outbreaks. This type of exercise also provided the best opportunity for evaluating the interoperability of the systems.

## Results

We carried out a global appraisal of the evaluations of 2SE FAG, PRISM and RMS. This made it possible to propose a new evaluation framework for evaluating military surveillance systems for the early detection of outbreaks on duty areas.

### Evaluations of 2SE FAG (table 2 and figure 4)

**Figure 4:**
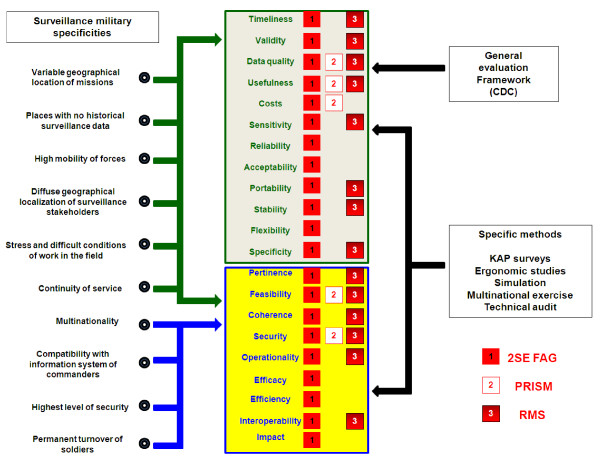
Schematic representation of the specificities of military surveillance and their connection with evaluation parameters (assessed by 2SE FAG = 1, by PRISM = 2, by RMS = 3) studied with the general evaluation framework and specific methods.

**Table 2:** Evaluation methods used for the French (2SE FAG) and British (PRISM and RMS) military syndromic surveillance systems and their main results

	**2SE FAG**	**PRISM**	**RMS**
**Evaluation programme**	Existing	Non-existent	Existing

**IE**			
**Methods**	Survey of data input personnel during deployment of the prototype (French Guiana, 2004)	Technical audit on a prototype (exercise in Oman, 1991)	Multinational exercise (Afghanistan, 2006)
**Actors**	2SE FAG team	PRISM team	RMS team SACT team
**Results**	Improvement of computerisation Adaptation of the training programme Building of the evaluation programme	Improved technical functioning	Development of analysis method

**INTE**			
**Methods**	Simulation exercise (2005) Ergonomic studies (2005–2006) KAP survey (2006) Multinational exercise (Afghanistan, 2006) Technical audits (2006–2007)	Evaluation of data input personnel activities	-
**Actors**	2SE FAG team ISPED SACT team	PRISM team	
**Results**	Continuous improvement process Evolution of recording software Cessation of PDA use in the field Development of analysis method Adaptation of indicators for commanders	Compliance rate of 10% for data input personnel	

**FE**			
**Methods**	CDC framework Simulation exercise	General audit (2004)	-
**Actors**	LSTM PRISM team	PRISM team 2SE FAG team	
**Results**	Lessons for generalisation of the system (2SE DJIB and others)	Lessons for development of RMS	

2SE FAG: "Système de surveillance en temps réel au sein des forces armées en Guyane"

2SE DJIB: Système de surveillance en temps réel au sein des forces françaises à Djibouti"

PRISM: Prototype Remote Illness and Symptom Monitor

RMS: Real-time Medical Surveillance

IE: initial evaluation, INTE: intermediate evaluation, FE: final evaluation

KAP: Knowledge, Attitude and Practice

SACT: Supreme Allied Command Transformation (NATO)

ISPED: *Institut de Santé Publique, Epidémiologie et Développement*

LSTM: Liverpool School of Tropical Medicine

Two military epidemiologists carried out an initial evaluation in October 2004. A survey, including standardised face-to-face interviews identified no initial system issues. Only 20% of health facilities were equipped with computers and only 12% of users were regularly using a PDA. This evaluation led to improvements in computerisation and the adoption of a training programme. A final evaluation programme was subsequently carried out.

Intermediate evaluations were carried out between 2004 and 2007. Two technical audits were performed in 2006 and 2007, an exercise simulating the release of an aggressive biological agent was carried out in 2005, ergonomics studies were carried out in 2005 and 2006, a KAP survey was carried out in 2006 and the multinational NATO "Disease Surveillance System Experiment" exercise was carried out in 2006, with British, American and Canadian partners. These evaluations were carried out by civilian and military personnel from organisations such as the *Institut de Santé Publique, d'Epidémiologie et de Développement *(ISPED) in Bordeaux, France, and the Supreme Allied Command Transformation (SACT) in Norfolk, USA. These evaluations, together with constant supervision, made possible the modification and regular improvement of the system. The frequency of PDA use was found to be 21.1%, and the use of such systems has now been ceased [14]. Stakeholders were knowledgeable, with 89.5% understanding the functioning and main objectives of the system, despite the dispensation of specific training to only 57% after arrival in French Guiana [14]. The recording software was considered easy to use by 73.7% of those questioned [15]. Extensive modifications were made to this software, the training program, the feedback system and the indicators supplied to commanders, as a direct result of these evaluations. The simulation exercise was used to evaluate timeliness, and demonstrated that an outbreak could be detected within 30 minutes. The NATO exercise also showed 2SE FAG to have a high level of interoperability, as demonstrated by the ease with which it was integrated into a multinational exercise network and sent French health data, in an appropriate format, to the multinational joint staff. This system was capable of detecting an anthrax outbreak within two hours and proposing appropriate measures to counter the disease [16]. This method of regular evaluation coupled with constant, rapid change has resulted in a dynamic system displaying continual operational improvement.

A final evaluation, based on the CDC framework, was carried out in 2007, in collaboration with the Liverpool School of Tropical Medicine (LSTM) and the Communicable Disease Control Unit of the Defence Medical Services Department (DMSD), UK [7]. Timeliness was shown to be excellent: the mean time between the first contact of a patient with the health system and the integration of his data into indicator format was estimated at 30 to 60 minutes. However, 16% of the stakeholders said that they did not carry out necessary tasks; acceptability therefore remains a serious issue. Assessments of validity showed that the system detected dengue fever outbreaks more effectively than malaria epidemics [7]. The usefulness of the system was demonstrated in 2006, during a major outbreak of dengue fever [17,18], but the lack of permanent system records made it impossible to quantify this usefulness. The reliability of the system was another problem, with 68% of stakeholders reporting that the system was sometimes unavailable, mainly for technical reasons. Based on this evaluation, the generalisation of the 2SE FAG system to all French military units on deployment was recommended. This evaluation also showed that the CDC framework was not entirely appropriate for the evaluation of military systems because it did not assess all the factors specifically important in a military context (e.g. interoperability, security, population turnover, high mobility, etc.). The proposed evaluation assesses these factors through other specific methods (figure 4).

### Results of evaluations of PRISM and RMS (table 2 and figure 4)

An initial evaluation of PRISM was carried out during an exercise in Oman in 2001, before the use of this system in a real armed forces deployment. This evaluation made it possible to improve the software, particularly for case recording. In 2003, PRISM was implemented within the UK contingent in Iraq, and 200 PDA were deployed. Data were transferred to the DSTL and then to the Communicable Disease Control Unit of the DMSD. No evaluation was performed in the field. After several months of use, an intermediate evaluation showed that only 10% of the users were actively using the system, as only 20 PDA had sent any data. Furthermore, the DSTL had no data-based threshold defining an alarm situation for a given symptom. The information generated was therefore of no operational use to commanders. PRISM was abandoned in 2004. A final evaluation was carried out to determine the causes of this failure. This evaluation was carried out by British and French partners, within the framework of a bilateral technical agreement for the development of military real-time surveillance. The poor acceptability of the system to military units was only one of several problems: these units had been deployed with no specific PRISM training programme, it was impossible for the organisers to supervise the use of the system during deployment in Iraq and the information supplied by the DSTL could not be used in its original format (list of number codes) and could not be used to generate operational indicators for commanders without significant formatting. It was therefore impossible for the Communicable Disease Control Unit of DMSD to analyse the data as this unit did not have the required biostatistical skills or available information. Many necessary improvements to system architecture, types and skills of stakeholders, tasks to be accomplished, and general system functioning were identified as a result.

RMS was set up in 2005, building on the experience gained from PRISM. A new architecture was defined, new partners, such as the Colindale Centre for Infections, joined the team and a new organisation concept was developed. Analytical capacities were developed, making it possible to generate operational information for viewing by commanders. This system is still being developed but was used in the NATO "Disease Surveillance System Experiment" exercise in 2006. During this exercise, this system took 24 hours to detect the anthrax outbreak, whereas the FR 2SE FAG system required only two hours [16].

### Proposal of a framework for evaluating military surveillance systems for the early detection of outbreaks on tours of duty

Our proposed new evaluation framework is summarised in figure 3. An initial evaluation is essential before or during the first deployment of a system or its prototype. At this point, coherence, pertinence, immediate acceptability, feasibility, and interoperability should be assessed. Those responsible for this evaluation must be deployed simultaneously with the system. The methods used in our evaluation framework are mostly surveys, with face-to-face interviews based on questionnaires and participation in a multinational exercise. This initial evaluation should be used to correct any problems identified and to create a formal evaluation program, with a detailed agenda.

Several intermediate evaluations should then be performed and any abnormalities should be rapidly corrected, to ensure continuous improvement. At this point, the dynamics of functioning, intermediate efficacy, operationality and coherence should be assessed. Various methods can be used for this assessment. A specific evaluation is required, focusing on those responsible for data recording, for which KAP surveys are ideal. Simulation exercises can be used to evaluate timeliness, sensitivity and specificity. Regular technical audits are required to check the functioning and back-up of the system and to ensure that the highest levels of security are maintained. Studies of ergonomics should focus on the enhancement of human-computer interfaces to improve their acceptability. At this point, participation in a multinational exercise would also be useful, for the assessment of interoperability. Military or civilian experts may be responsible for these evaluations.

The final evaluation is essential. It should be carried out at the end of a prototype phase (as with 2SE FAG), at the end of deployment in a theatre of action or when a system ceases to be used (as with PRISM). At this point, efficacy, timeliness, validity, sensitivity, specificity, data quality, usefulness, portability, stability and reliability should be assessed. A study of costs, efficiency, impact and interoperability should also be carried out. The reference method for the final evaluation is the CDC framework, adapted to the specific characteristics of the military environment. If a deployment or mission is too long, this method could also be used after several months or years of functioning. A multinational exercise could again be useful at this stage. The final evaluation is expected to generate conclusions and recommendations allowing the generalisation of the system or allowing its use for another deployment, following adoption of the proposed corrections and improvements.

## Discussion

The French and British armed forces have acquired expertise in the area of syndromic surveillance, through their 2SE FAG, PRISM and RMS projects. The evaluation of these projects has become a priority, because only a valid evaluation method can provide useful information for the generalisation of such systems to all armed forces on duty areas.

The two countries have had different experiences in terms of evaluation. The UK armed forces developed their system without a viable evaluation program. As a result, it was not possible to identify and correct anomalies. This resulted in the failure of the PRISM system. If a robust evaluation method had been implemented during the development of PRISM, the system might have been more successful. However, the final evaluation did lead to the identification of errors and lessons were learnt from this experience, improving the development of RMS. The evaluation of RMS was incorporated into the development programme from the start, and evaluations will be carried out for each stage of development. The use of RMS in the NATO exercise demonstrated that these methods have led to considerable improvement.

French experts were able to analyse the failure of PRISM before embarking on their own project, because the British and French projects were not developed over the same time period. The French team considered evaluation to be a major issue from the outset. Since the start of 2SE FAG development in 2002, a structured evaluation method has been used to ensure continuous improvement to the system, with unbiased observers evaluating the value of this surveillance system to the armed forces. Many civilian experts have therefore been involved in the operational evaluation of 2SE FAG, making it possible to modify and adapt the system. Without these methods, 2SE FAG might have suffered the same fate as PRISM. The 2SE FAG prototype phase has just been completed and, the usefulness of this system having being demonstrated, the Joint Staff has decided to continue its use within the armed forces in French Guiana and to install this system in Djibouti, under the name 2SE DJIB. All this work is a part of a more general system: *Alerte et surveillance en temps reel *(ASTER). Evaluation has played a major role in improvements to 2SE FAG and the creation of 2SE DJIB. The method we propose here is currently being used to evaluate 2SE DJIB.

The framework proposed in 2004 by the CDC [4] has proved a useful tool. Several American and British teams have evaluated their systems with this framework, and have produced recommendations to improve functioning, as for ESSENCE [19] and NHS Direct [20]. This framework has also been used to evaluate 2SE FAG [21], but was found to be of limited use in this cases, as it was not adapted to the specific needs of the armed forces on duty. We propose new tools and studies for evaluating all aspects of the use of this system in armed forces on deployment.

The lack of epidemics due to bioterrorism agents limits opportunities for evaluating syndromic surveillance systems. Outbreaks of natural agents can be studied, such as dengue and malaria for 2SE FAG and influenza for other systems [22,23]. Alternatively outbreaks due to aggressive agents may be simulated, as for 2SE FAG and RMS. Such simulations have already been carried out, for anthrax, using a specific epidemic simulation model [24]. Other simulation models have been developed, including one simulating an outbreak of anthrax due to inhalation after the release of this agent in an aerosol [25]. Simulation has also been used to compare statistical methods for early detection, such as the "SaTScan trade mark" and "small area regression and testing scores" (SMART) methods [26].

Another important issue is collaboration with external partners. The active involvement of the French armed forces in the evaluation of PRISM, the participation of SACT in the evaluation of both 2SE FAG and RMS, and the participation of ISPED, LSTM and DMSD in the evaluation of 2SE FAG have all led to the overall improvement of these systems.

The involvement of commanders in the evaluation programme is a major issue. Each evaluation process costs money and takes time, and therefore represents a significant burden. The surveys are impossible to organise without authorisation from the commanders, who must therefore understand the benefits of operational surveillance systems providing essential information outlining the most appropriate course of action in the theatre of action. The challenge is to provide non-medical decision-makers with appropriate information, in a form that is easy to understand and can be used directly. This has been achieved with the 2SE FAG indicators (with coloured indicators for alarm level, risk maps and a summary of the main results), which are updated in real time, but not with PRISM. This evaluation system is of potential interest to the entire public health community, as it can present public health information in a format that individuals from outside the field of public health can understand and act on in an informed manner.

The proposed evaluation method provides a complete, global evaluation of military syndromic surveillance systems. Additional methods could be included for the evaluation of specific parts of the system. For example, a recent study evaluated the utility of ICD 9 codes for the ESSENCE system [27].

## Conclusion

This proposed evaluation method should allow other countries potentially interested in the development of real-time surveillance within their armed forces to benefit from the experience of the British and French armed forces. As it is impossible to predict the time and the location of future deployments, this evaluation method was designed to be used directly.

Evaluation remains the weakest link in the public health chain. The method proposed here makes it possible to improve military surveillance systems, which have been shown to be essential. It could be also useful for mobile civilian systems or for national security surveillance systems.

It should be borne in mind that real-time surveillance systems are simply a source of information, raising the alarm and assisting decision-makers in the complex task of managing the health situation. Surveillance is only one of a battery of decision-making aids for medical experts. Others include medical intelligence, epidemiological investigations and prediction of the progression of the phenomena observed. All these decision support systems require improvement through rigorous evaluation.

## Competing interests

The authors declare that they have no competing interests.

## Authors' contributions

JBM co-ordinated the 2SE FAG evaluation programme, and took part in each stage of this programme and in those for PRISM and RMS. He had the idea of developing the new framework. HC took part in the ergonomics study and the final evaluation of 2SE FAG. ADG conducted the evaluations of PRISM and RMS. He was involved in the final evaluation of 2SE FAG. HLJ conducted the final evaluation of 2SE FAG. GT participated in the initial and intermediate evaluations of 2SE FAG. DW participated at all the evaluation steps for PRISM and RMS. BD was responsible of the technical audits of 2SE FAG and took part in the entire programme. JBP was responsible for the entire 2SE FAG evaluation programme and took part in the evaluation of PRISM and RMS. All the authors read and approved the final manuscript.

## Pre-publication history

The pre-publication history for this paper can be accessed here:


